# Ginsenoside Rg3 Improves Cardiac Function after Myocardial Ischemia/Reperfusion via Attenuating Apoptosis and Inflammation

**DOI:** 10.1155/2016/6967853

**Published:** 2016-12-26

**Authors:** Li-ping Zhang, Yi-chuan Jiang, Xiao-feng Yu, Hua-li Xu, Min Li, Xue-zhong Zhao, Da-yuan Sui

**Affiliations:** ^1^Departments of Cardiovascular Medicine, First Hospital, Jilin University, Changchun 130021, China; ^2^Departments of Pharmacology, College of Pharmacy, Jilin University, Changchun 130021, China

## Abstract

*Objectives*. Ginsenoside Rg3 is one of the ginsenosides which are the main constituents isolated from* Panax ginseng*. Previous study demonstrated that ginsenoside Rg3 had a protective effect against myocardial ischemia/reperfusion- (I/R-) induced injury.* Objective*. This study was designed to evaluate the effect of ginsenoside Rg3 on cardiac function impairment induced by myocardial I/R in rats.* Methods*. Sprague-Dawley rats were subjected to myocardial I/R. Echocardiographic and hemodynamic parameters and histopathological examination were carried out. The expressions of P53, Bcl-2, Bax, and cleaved caspase-3 and the levels of TNF-*α* and IL-1*β* in the left ventricles were measured.* Results*. Ginsenoside Rg3 increased a left ventricular fractional shortening and left ventricular ejection fraction. Treatment with ginsenoside Rg3 also alleviated increases of left ventricular end diastolic pressure and decreases of left ventricular systolic pressure and ±dp/dt in myocardial I/R-rats. Ginsenoside Rg3 decreased apoptosis cells through inhibiting the activation of caspase-3. Ginsenoside Rg3 also caused significant reductions of the contents of TNF-*α* and IL-1*β* in left ventricles of myocardial I/R-rats.* Conclusion*. The findings suggested that ginsenoside Rg3 possessed the effect of improving myocardial I/R-induced cardiac function impairment and that the mechanism of pharmacological action of ginsenoside Rg3 was related to its properties of antiapoptosis and anti-inflammation.

## 1. Introduction

The prevalence of heart failure reaches 1% of the population aged over 65 years in the world. It estimated that 37% of all cases of symptomatic heart failure worldwide were caused by myocardial ischemia [1]. The cardiomyocytes are responsible for contractile function. However, the cardiomyocytes have a limited capacity to regenerate. Therefore, it is important to preserve existing cardiomyocytes to support a proper function. Myocardial infarction causes an ischemia of the territory, which will lead to a death of up to a billion cardiomyocytes [2]. Apoptosis, a distinct form of cell death, is a main mechanism of cardiomyocyte loss during myocardial ischemia. There is a heightened apoptosis in the peri-infarct and noninfarcted cardiomyocyte, with an increase of left ventricular diameter and decrease of cardiac function. Therefore, therapy inhibiting apoptosis of cardiomyocyte is a promising approach to improve cardiac function after myocardial infarction [3, 4]. Ischemic cardiomyocytes also trigger an intense inflammatory reaction. A large body of experimental evidences demonstrate that inflammation aggravates ischemic injury [5, 6].

Ginseng, the root of* Panax ginseng* C.A. Meyer listed in the Chinese Pharmacopoeia, is widely used to treat cardiovascular disorders. Ginsenosides are the bioactive ingredients of ginseng. Studies indicated that ginsenosides had potential cardiovascular benefits through many mechanisms including the properties of antioxidation and anti-inflammation [7]. Ginsenoside Rg3 is one of the ginsenosides. It has been reported that ginsenoside Rg3 improved cardiac functions by regulating mitochondria dynamic remodeling and enhancing the quantity and quality of mitochondria [8]. Recent study demonstrated that ginsenoside Rg3 attenuated myocardial ischemia/reperfusion (I/R) injury via Akt/endothelial nitric oxide synthase signaling pathway [9]. Up to now, most studies have focused on the protective effects of ginsenoside Rg3 on myocardial I/R injury. However, the potential ability of ginsenoside Rg3 to improve cardiac function after myocardial I/R has not yet been investigated. Therefore, the present study aims to evaluate whether ginsenoside Rg3 could improve cardiac function after myocardial I/R in rats.

## 2. Materials and Methods

### 2.1. Materials

**Figure 1:**
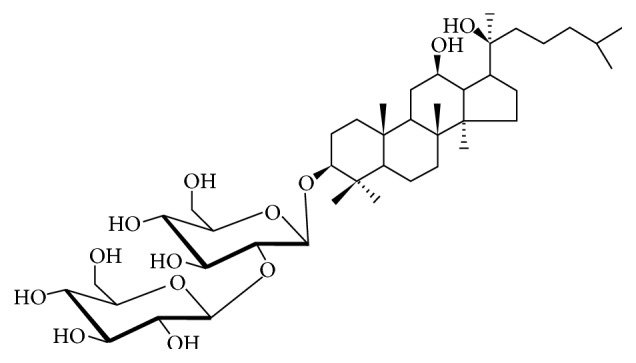
The chemical structure of ginsenoside Rg3.

Ginsenoside Rg3, a white amorphous powder, was provided by Department of Medicinal Chemistry, Jilin University. The chemical structure of ginsenoside Rg3 was shown in Figure 1. The molecular weight of ginsenoside Rg3 was 785. Its purity (>99%) was determined by HPLC. TNF-*α* and IL-1*β* enzyme-linked immunosorbent assay (ELISA) kits were purchased from R&D Systems, Inc. (Minneapolis, MN, USA).

### 2.2. Animals

Male Sprague-Dawley rats weighing 270–290 g were obtained from the Experimental Animal Center of Jilin University. They were housed in a well-ventilated animal unit (22 ± 2°C, 12-h light/dark cycle) and had free access to a standard diet. The rats were given water ad libitum. The experiments were carried out according to the Guide for the Care and Use of Laboratory Animals published by the National Institutes of Health (NIH publication number 85-23, revised 1996) and were approved by the local Ethics Committee of Jilin University (J130926).

### 2.3. Experimental Protocols

The rats were anesthetized with chloralose (300 mg/kg, intraperitoneally). Myocardial ischemia was produced via one-stage occlusion of the left coronary artery for 30 min followed by reperfusion as described previously [10]. The rats in the sham group (sham) were subjected to the same procedure without the left anterior descending coronary artery ligation. Myocardial I/R-rats were randomly divided into three groups as follows: myocardial ischemia/reperfusion group (I/R), ginsenoside Rg3 (5 mg/kg) group, and ginsenoside Rg3 (20 mg/kg) group. At the time point of reperfusion, rats in the sham and I/R groups were intragastrically administered with normal saline. Animals of ginsenoside Rg3 groups were intragastrically treated with ginsenoside Rg3 at doses of 5 or 20 mg/kg, once a day for 7 days.

### 2.4. Echocardiography

At 2 h after last treatment, transthoracic echocardiography was performed as previously described using a standard setting with a 10S scan head (GE Vivid I, GE Healthcare, USA). Animals were anesthetized with urethane (1 g/kg, intraperitoneally). Two-dimensional and M-mode echocardiographic measurements were conducted. A short-axis two-dimensional image of the left ventricle was first obtained at the position of the papillary muscles. Then, M-mode images were acquired at a sweep speed of 100 mm/s and stored digitally. After the left ventricular internal dimension at diastole (LVIDd) and left ventricular internal dimension at systole (LVIDs) were acquired from M-mode images, left ventricular fractional shortening (FS) and left ventricular ejection fraction (EF) were calculated automatically by the equipment. The parameters were measured by one experienced echocardiographer who was blind to the treatment.

### 2.5. Hemodynamic Measurements

After echocardiography measurement, the right common carotid artery of the rats was cannulated with a 2 F polyethylene catheter into the left ventricle. Thereby, left ventricular systolic pressure (LVSP), left ventricular end diastolic pressure (LVEDP), and positive and negative maximal values of the first derivative of left ventricular pressure (±dp/dt) were measured using a hemodynamic analyzing system (Model RM-6000, Nihon Kohden, Japan).

### 2.6. TUNEL Staining

TUNEL staining was assessed by the In Situ Cell Death Detection Kit, POD (Roche Ltd., Basel, Switzerland). After the cardiac function evaluation, the sections of rat hearts were prepared. The staining was performed according to the protocol provided by the manufacturer. Ten fields in each section were randomly selected for apoptotic cell counting in a blinded manner using an Olympus IX51-reflected light fluorescence microscope (Olympus Corporation, Tokyo, Japan). Then the apoptosis cells were calculated.

### 2.7. Western Blot Analysis

The protein of the left ventricles was separated by sodium dodecyl sulfate-polyacrylamide gel electrophoresis and then transferred to the polyvinylidene difluoride membranes using an electrophoretic transfer system. The membranes were blocked for 2 h with 5% bovine serum albumin and incubated for 24 h at 4°C with primary antibody. The primary antibodies were as follows: P53 (1 : 1000, Cell Signaling Technology, Danvers, MA, USA), Bcl-2 (1 : 500, Abcam, Cambridge, UK), Bax (1 : 500, Abcam, Cambridge, UK), cleaved caspase-3 (1 : 1000, Abcam, Cambridge, UK), and *β*-actin (1 : 2000, ZSGB-BIO, Beijing, China). The primary antibodies were detected with horse radish peroxidase-conjugated goat anti-mouse or goat anti-rabbit secondary antibody (1 : 2000). ECL detection reagents were used to detect the binding. For the quantitative analysis of the density of the immunoblot bands, densitometry was performed with the Gel-Pro Analyzer Version 3.0.

### 2.8. Assays of TNF-*α* and IL-1*β* in the Left Ventricles

The supernatants of the left ventricles were prepared as follows. Five hundred milligrams of the left ventricles was homogenized in 9 volumes of ice-cold saline and centrifuged (1000 ×g) at 4°C for 15 min. The supernatants were removed and stored at −80°C for further analysis. The amount of protein in the supernatant was measured according to the previous method [11] using bovine serum albumin as standard. The levels of TNF-*α* and IL-1*β* in supernatants of the left ventricles were estimated with an ELISA plate reader, using commercially available ELISA kits and following the manufacturer's protocols. Duplicate samples were analyzed for each sample.

### 2.9. Statistical Analysis

SPSS 22.0 Statistical Software was used for the analysis. The data are expressed as the mean ± SD, and the statistical significance of the data was determined using one-way analysis of variance (ANOVA) followed by Dunnett's test. *P* value < 0.05 was considered as statistically significant.

## 3. Results

### 3.1. Effect of Ginsenoside Rg3 on LVIDd, LVIDs, FS, and EF

**Figure 2:**
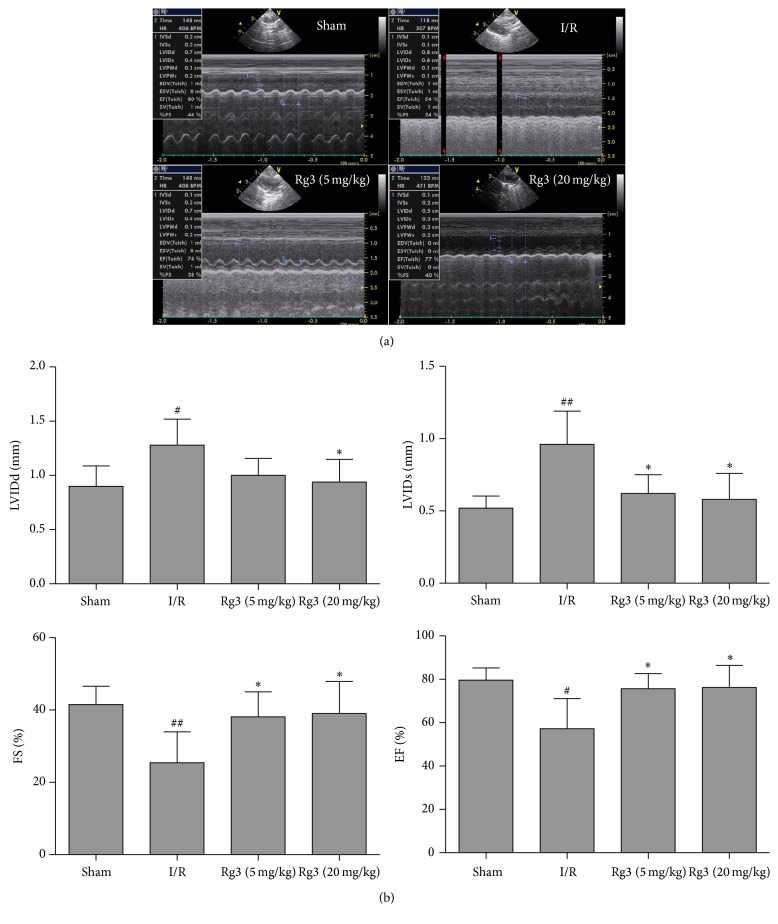
Effect of ginsenoside Rg3 on LVIDd, LVIDs, FS, and EF. (a) Representative echocardiographic M-mode records. (b) Ginsenoside Rg3 treatment improves cardiac function with lowered LVIDd and LVIDs and increased EF and FS. LVIDd, left ventricular internal dimension at diastole; LVIDs, left ventricular internal dimension at systole; FS, left ventricular fractional shortening; EF, left ventricular ejection fraction; Sham, sham group; I/R, myocardial ischemia/reperfusion group; Rg3 (5 mg/kg), ginsenoside Rg3 at dose of 5 mg/kg group; Rg3 (20 mg/kg), ginsenoside Rg3 at dose of 20 mg/kg group. The data are expressed as the mean ± SD, *n* = 11-12; ^#^*P* < 0.05 versus the sham group; ^##^*P* < 0.01 versus the sham group; ^*∗*^*P* < 0.05 versus the I/R group.

We evaluated the effect of ginsenoside Rg3 on cardiac function using echocardiography. As shown in Figure 1, compared with sham group, the myocardial I/R-rats showed increases of LVIDd (1.28 ± 0.23 versus 0.90 ± 0.18 mm) and LVIDs (0.96 ± 0.23 versus 0.52 ± 0.08 mm). Moreover, FS (25.42 ± 8.52% versus 41.53 ± 5.04%) and EF (57.24 ± 13.84% versus 79.64 ± 5.59%) in the I/R group were significantly reduced when compared with the sham group. However, ginsenoside Rg3 treatment improved cardiac function with lowered LVIDd and LVIDs and increased EF and FS as compared to the I/R group (Figures 2(a) and 2(b)).

### 3.2. Effect of Ginsenoside Rg3 on LVSP, LVEDP, and ±dp/dt

**Figure 3:**
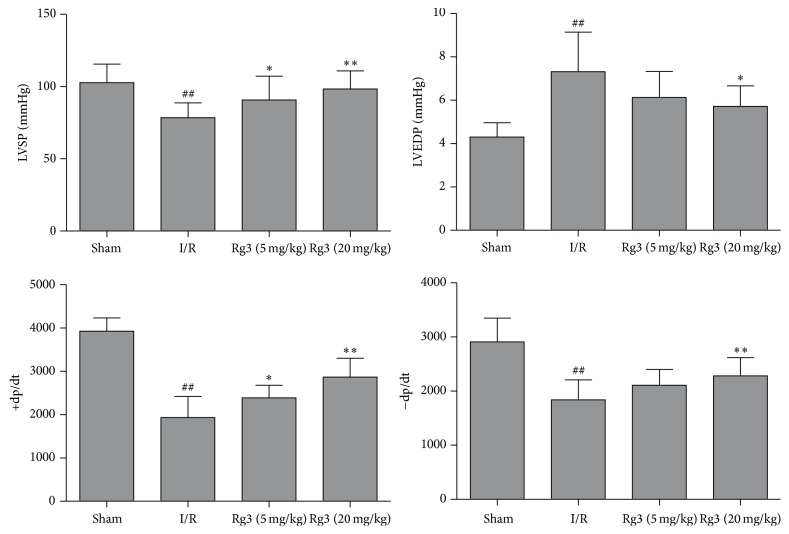
Effect of ginsenoside Rg3 on LVSP, LVEDP, and ±dp/dt. LVSP, left ventricular systolic pressure; LVEDP, left ventricular end diastolic pressure; Sham, sham group; I/R, myocardial ischemia/reperfusion group; Rg3 (5 mg/kg), ginsenoside Rg3 at dose of 5 mg/kg group; Rg3 (20 mg/kg), ginsenoside Rg3 at dose of 20 mg/kg group. The data are expressed as the mean ± SD, *n* = 11-12; ^##^*P* < 0.01 versus the sham group; ^*∗*^*P* < 0.05 or ^*∗∗*^*P* < 0.01 versus the I/R group.

Compared with the sham group, myocardial I/R induced cardiac function impairment characterized by a significant increase of LVEDP and decreases of LVSP and ±dp/dt. Treatment with ginsenoside Rg3 at doses of 5 or 20 mg/kg alleviated the increase of LVEDP and the decreases of LVSP and ±dp/dt induced by myocardial I/R (*P* < 0.05 or *P* < 0.01, Figure 3).

### 3.3. Effect of Ginsenoside Rg3 on Apoptotic Cells

**Figure 4:**
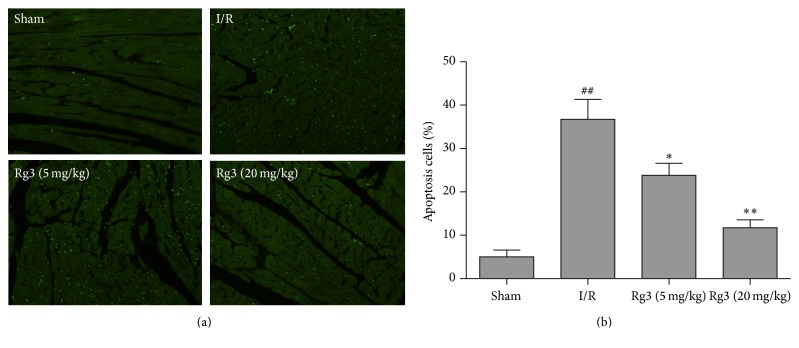
Effect of ginsenoside Rg3 on apoptosis cells. (a) Apoptotic cardiomyocytes were identified by TUNEL analysis. TUNEL-positive cells were manifested as a marked appearance of green apoptotic cell nuclei (400x). (b) Treatment with ginsenoside Rg3 significantly reduced the number of apoptosis cells. Sham, sham group; I/R, myocardial ischemia/reperfusion group; Rg3 (5 mg/kg), ginsenoside Rg3 at dose of 5 mg/kg group; Rg3 (20 mg/kg), ginsenoside Rg3 at dose of 20 mg/kg group. The data are expressed as the mean ± SD, *n* = 3; ^##^*P* < 0.01 versus the sham group; ^*∗*^*P* < 0.05 or ^*∗∗*^*P* < 0.01versus the I/R group.

To evaluate the effect of ginsenoside Rg3 on the apoptosis of cardiomyocyte, TUNEL staining was carried out at the surrounding infarction areas of the left ventricles. The representative images were showed in Figure 4(a). TUNEL-positive cells were manifested as a marked appearance of green apoptotic cell nucleus. Apoptosis cells markedly increased in the I/R group when compared with that in the sham group. Treatment with ginsenoside Rg3 significantly reduced the number of apoptotic cells (*P* < 0.05 or *P* < 0.01, Figure 4(b)).

### 3.4. Effect of Ginsenoside Rg3 on the Expressions of P53, Bcl-2, Bax, and Cleaved Caspase-3

**Figure 5:**
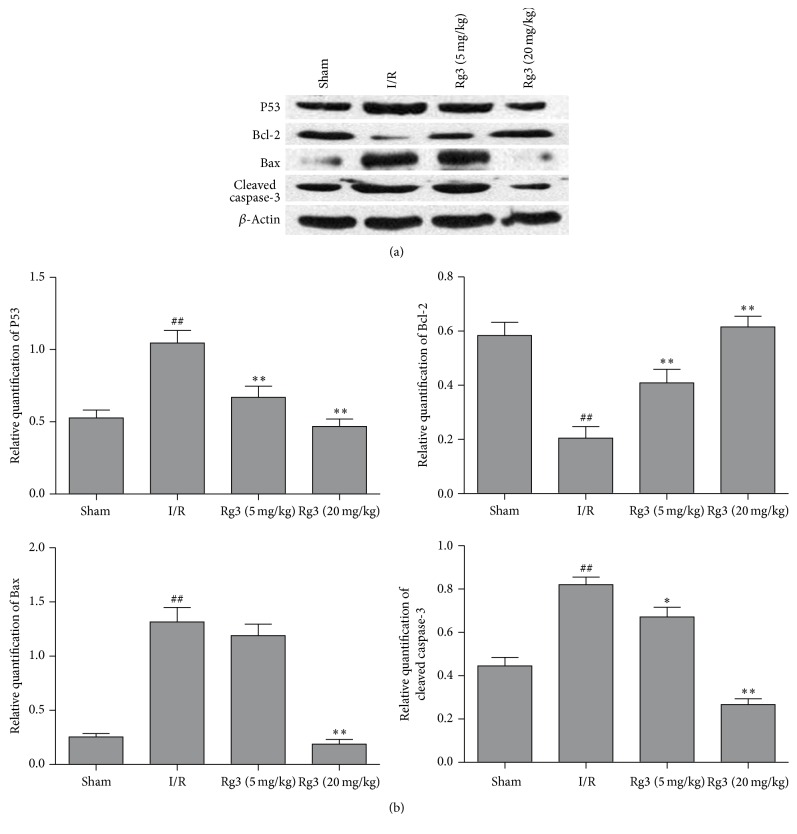
Effect of ginsenoside Rg3 on the expressions of P53, Bcl-2, Bax, and cleaved caspase-3. (a) Representative immunoblotting bands of P53, Bcl-2, Bax, and cleaved caspase-3. (b) Ginsenoside Rg3 reduced the levels of P53, Bax, and cleaved caspase-3 and augmented the expression of Bcl-2. Sham, sham group; I/R, myocardial ischemia/reperfusion group; Rg3 (5 mg/kg), ginsenoside Rg3 at dose of 5 mg/kg group; Rg3 (20 mg/kg), ginsenoside Rg3 at dose of 20 mg/kg group. The data are expressed as the mean ± SD, *n* = 3; ^##^*P* < 0.01 versus the sham group; ^*∗*^*P* < 0.05 or ^*∗∗*^*P* < 0.01 versus the I/R group.

As shown in Figure 4, protein expression of P53, Bax, and cleaved caspase-3 increased, whereas protein expression of Bcl-2 decreased following myocardial I/R. Ginsenoside Rg3 treatment reduced the levels of P53, Bax, and cleaved caspase-3 and augmented the expression of Bcl-2 (*P* < 0.05 or *P* < 0.01, Figures 5(a) and 5(b)).

### 3.5. Effect of Ginsenoside Rg3 on Levels of TNF-*α* and IL-1*β* in the Left Ventricles

**Figure 6:**
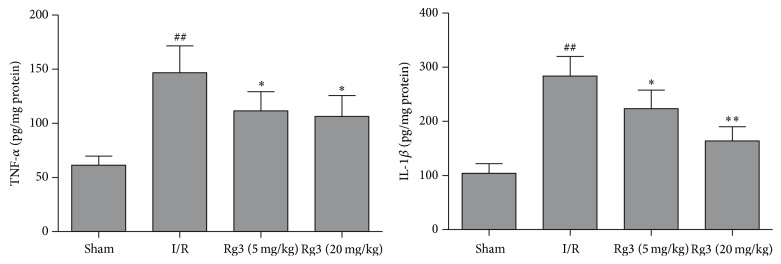
Effect of ginsenoside Rg3 on levels of TNF-*α* and IL-1*β* in the left ventricles. Sham, sham group; I/R, myocardial ischemia/reperfusion group; Rg3 (5 mg/kg), ginsenoside Rg3 at dose of 5 mg/kg group; Rg3 (20 mg/kg), ginsenoside Rg3 at dose of 20 mg/kg group. The data are expressed as the mean ± SD, *n* = 5-6; ^##^*P* < 0.01 versus the sham group; ^*∗*^*P* < 0.05 or ^*∗∗*^*P* < 0.01 versus the I/R group.

The levels of TNF-*α* and IL-1*β* in the left ventricles of the I/R group increased when compared with that of the sham group (*P* < 0.01). However, treatment with ginsenoside Rg3 at doses of 5 and 20 mg/kg markedly inhibited the elevation of levels of TNF-*α* and IL-1*β* induced by myocardial I/R (*P* < 0.05 of *P* < 0.01, Figure 6).

## 4. Discussion

The incidence of myocardial infarction has decreased over the last two decades in developed countries. Because it finally developed into heart failure, myocardial infarction remained the major killer during the past decade [12]. Although existing therapies have improved the clinical course of heart failure patients, new approaches are urgently needed to enhance the quality of life and reduce the morbidity and mortality of myocardial infarction patients. The major findings of this study are as follows. First, ginsenoside Rg3 improved myocardial I/R-induced cardiac function impairment, as demonstrated by the increases of the FS, EF, LVSP, and ±dp/dt and the decrease of the LVEDP. Second, evidence from these experiments indicated that the mechanism of pharmacological action of ginsenoside Rg3 was related to its properties of antiapoptosis and anti-inflammation.

Cardiac functions are closely associated with the ventricular systolic and diastolic functions. Studies have demonstrated that myocardial I/R injury may induce the changes of cardiac hemodynamic parameters and then affect cardiac function [13]. Echocardiography and hemodynamic analyzing system are widely used for measuring cardiac function in myocardial ischemic animal model [14, 15]. In our experiments, the LVIDs, FS, and EF decreased while LVIDd increased in myocardial I/R-rats, indicating that myocardial I/R impaired cardiac function. However, the changes of LVIDs, FS, EF, and LVIDd were ameliorated by ginsenoside Rg3 treatment. In line with these results, the data of hemodynamic measurements also showed that ginsenoside Rg3 alleviated the increase of LVEDP and the decreases of LVSP and ±dp/dt induced by myocardial I/R. These findings suggested that ginsenoside Rg3 could improve cardiac function after myocardial I/R injury.

After myocardial ischemia, apoptosis plays a critical role in both mechanical and molecular mechanisms of cardiac dysfunction [16]. In the present study, TUNEL assay was used to evaluate the level of myocardial apoptosis. The apoptotic cardiomyocytes increased in rat hearts subjected to myocardial I/R. Treatment with ginsenoside Rg3 resulted in a reduction of cardiomyocyte apoptosis rate, indicating that the antiapoptotic effect of ginsenoside Rg3 contributes to the improvement of cardiac function. Caspase-3 plays a key role in cell apoptosis. Caspase-3 is activated by a series of signal transduction cascades. Bcl-2 family members play important roles in regulating apoptotic signaling. Bcl-2 family members are divided into two subgroups. The first group is the antiapoptotic protein, such as Bcl-2 and Bcl-x. The second group promote apoptosis including Bax and Bak [17]. P53 controls the expressions of a good many of genes involved in apoptosis. P53 is involved in both intrinsic and extrinsic pathways of apoptosis. It induces the transcription of several proteins like Bax and Bid, which is called transcription-dependent apoptotic pathway [18]. Myocardial ischemic injury triggers TNF-*α* release from the cardiomyocyte. The secreted TNF-*α* further stimulates the release of proinflammatory cytokines from infiltrating leukocytes and endothelial cells, initiating the cytokine cascade [19, 20]. TNF-*α* and IL-1*β* are contributors to cardiac dysfunction. The present study revealed that treatment with ginsenoside Rg3 significantly inhibited the increases of P53, Bax, and cleaved caspase-3 and the decrease of Bcl-2 induced by myocardial I/R. It is showed that ginsenoside Rg3 also blocked the increases of TNF-*α* and IL-1*β* in myocardial I/R-rats. Therefore, these findings indicated that the mechanism of pharmacological action of ginsenoside Rg3 was related to its properties of antiapoptosis and anti-inflammation.

There are limitations in this study. Although apoptosis has been considered as a noninflammatory mode of cell death, recent studies have provided evidences that apoptotic cells are invariably noninflammatory, particularly in the context of apoptosis induced by the members of the TNF family. Cells undergoing apoptosis is also associated with the production of a range of proinflammatory cytokines and chemokines by the dying cell [21]. However, this study did not elucidate whether there are any relationships between the effects of antiapoptosis and anti-inflammation of ginsenoside Rg3. Further studies are needed to verify this important issue.

## 5. Conclusions

In conclusion, the findings of the present study indicated that ginsenoside Rg3 possessed the effect of improving myocardial I/R-induced cardiac function impairment and that the mechanism of pharmacological action was related to its properties of antiapoptosis and anti-inflammation. Therefore, the possible therapeutic application of ginsenoside in myocardial infarction patients at risk for heart failure is worth researching.
